# Moisture Transport in Loose Fibrous Insulations under Steady-State Boundary Conditions

**DOI:** 10.3390/ma16247656

**Published:** 2023-12-15

**Authors:** Piotr Kosiński

**Affiliations:** Faculty of Geoengineering, University of Warmia and Mazury in Olsztyn, ul. Heweliusza 10, 10-724 Olsztyn, Poland; piotr.kosinski@uwm.edu.pl; Tel.: +48-89-523-3350

**Keywords:** sorption behavior, loose-fill insulation materials, natural fiber-based materials, moisture buffering in materials, thermal conductivity

## Abstract

This research aimed to compare the transport capacity of loose-fill mineral wool, cellulose fibers, and wood wool to transfer moisture under steady-state conditions. The tests were carried out in the heat flow meter apparatus, which created a constant thermal field, limiting samples of sorptive moistened materials. The thermal conductivity, stabilization time, and moisture content of the samples were measured. Based on the variation in the results, the dynamism of moisture transport in the materials was determined. Mineral wool samples showed the lowest sorption. As a result, the moisture transport in this material stopped the fastest. In the case of cellulose and wood fibers, moisture transport continued throughout the whole test procedure. It was noted that the amount of moisture transport is influenced primarily by the structure of the fibers, the moisture content, and the possible presence of air in the pores. The wetter the material, the faster the transport. The dynamism of moisture transport according to trends of thermal conductivity changes over time was analyzed. The greater the slope of the linear regression line, the greater the dynamics of change. The smallest dynamics of change were found for mineral wool, for which the measured slope was between −0.008 and −0.033. For cellulose and wood wool, the range of slope was from −0.141 to −0.210, and from −0.162 to −0.211, respectively. The results of this research may provide the basis for further work on buffering moisture in the adjacent internal layers of the frame walls.

## 1. Introduction

Fibrous insulation materials are susceptible to transporting, buffering, and evaporating moisture in a dry environment. Each fibrous material has a different ability to transport moisture, absorb it, or dry it. These properties depend on the unique structure of the fibers, their arrangement, and their density. However, what is common to all fibrous materials is that moisture transport changes their thermal insulation properties. Michalkova and Durica described the exploitation problems of thermal properties of glass and basalt fibers, sheep wool, and wood fiber boards inbuilt in the wood frame test house in Zilina. After a few years of exploitation, the thermal conductivity of wood fiber insulation raised by 90% as the effect of moisture accumulated. However, a frame filled with sheep wool did not reveal an accumulated moisture problem. The insulations made of glass or basalt wool were almost dry [1]. Georgescu et al. investigated frame wall elements filled with reed straw. They found that the water evaporation phenomenon, along with the increase in humidity inside the air cavities, increased the thermal conductivity coefficient values because of the more intense movement of the humid air and convection occurrence [2]. Vrana and Gudmundsson compared cellulose and stone wool in terms of moisture properties resulting from condensation and ice formation. They noted an effect on the growth of the moisture resistance factor for temperature fields below the freezing point: for cellulose, it was about 60%, while for mineral wool it was almost 300%. Despite this, both materials remained highly permeable during the tested period, despite ice formation in the structure [3].

The accumulation and distribution of moisture content in fibrous insulations is a complex combination of moisture absorption, condensation, and liquid water movement. Mass transport in fibrous materials can be due to several different modes. The three primary modes are molecular diffusion for gases, capillary for liquids, and pressure-induced convection. Convection is a moisture risk in an unvented assembly of fibrous insulation materials [4,5,6,7]. Moisture distribution in natural origin materials is comprehensively described in the case of wood. The redistribution of the moisture induced by thermal gradients results in a transient heat flow. During redistribution, the apparent conductivity is larger than the steady-state conductivity [8]. While MacLean observed a long time ago that in solid wood elements of 0.5 to 0.75-inch thickness with a moisture content below 15%, most of the moisture redistribution took place within 24 h without significant change in conductivity. After that, with rising moisture content, even over 60%, the redistribution took several days to reach the final state [9]. On the other hand, Bomberg and Shirtliffe observed a similar effect in the mineral elements [10]. The researchers stated that the final condition was not a true steady-state equilibrium because of opposing flows of water vapor and bound water. Moisture distribution in fibrous insulation materials is widely analyzed in Scandinavian countries and mainly concerns the risk of mold development. Much attention has also been paid to increased convection as the driving force of moisture transport in thick layers of fibrous insulation in roofs and walls. Convection flows caused by air filtration change the moisture distribution, often increasing it [11,12,13,14,15,16]. The requirements for the thermal protection of buildings in European countries influence the use of thicker thermal insulation of walls and roofs. Thus, the topics discussed so far in cold regions are also starting to be widely commented on in countries with a moderate climate [17,18].

Moisture-inducing heat transfer was also analyzed in the sandwich fibrous insulations by Leskovšek and Medved [19] and Chauhan et al. [20]. Even a small mass of water in the insulation matrix can result in a significantly increased average heat flux through the sandwich panel. Laboratory studies of the impact of moisture content on the thermal conductivity of building materials are usually performed using transient methods. Bal et al. used a modified hot plate stand (pulse heat flux generation) to measure the thermal conductivity of wet composite boards made of laterite-based bricks with millet waste additive [21]. A similar method was used by Damfeu et al. to measure the thermal conductivity of the cinder block of pozzolans and the cinder block of sand, with regards to the moisture content of the samples [22]. The principles of this method were described by Jannot et al. [23]. Szymczak-Graczyk et al. used a hot wire method to measure the moisture influence on the thermal conductivity of perlite concrete, PU foam, and expanded clay [24]. Collet and Pretot investigated the thermal conductivity of wet hemp concrete elements using the hot wire method. As they stated, the main advantage of this method, compared to steady-state methods like the hot plate, is that it is a transient method that does not induce (or that does limit) water migration during the test [25]. Prałat et al. utilized the hot wire method to measure the thermal conductivity of moistened gypsum modified with a cellulose-based polymer and aerogels [26]. Yang et al. utilized the hot disk method to measure thermal conductivity under different moisture contents of polystyrene, aerogel, PUR, cotton, and rock wool [27].

On the other hand, researchers also utilized a stationary method to assess the thermal conductivity of materials under different levels of moisture content. Abdou and Budaiwi tested fiberglass, rock wool, and mineral wool using the Holometrix heat flow meter [28]. Gaujena et al. measured the thermal conductivity of different moisture contents of the hemp fiber insulations with the heat flow meter apparatus FOX 600 [29]. Another technique called the ‘Boxes method’ was presented by Taoukil et al. to measure the thermal conductivity of a wood–concrete composite under different moisture contents. This method is similar to the widely known stationary heat flow meter techniques [30]. Another stationary technique was used by Dias and Delkumburewatte to measure the thermal conductivity of a knitted structure under different moisture content levels. A tested fabric was placed over a combination of heating matt, copper, and nylon plates. The assumption of the method was that the heater mat continuously generated the same heat energy [31]. The results of all of the investigations showed the rising tendency of thermal conductivity with rising moisture content. Both methods, stationary and non-stationary, are commonly in use to determine the moisture influence on the thermal conductivity of building materials. Almost 40 years ago, Langlasis and Klarsfeld concluded their eight years of work in the laboratory on fibrous insulation. They stated that the phenomenon of thermomigration makes it impossible to plot a single curve for a material predicting the variation in thermal conductivity versus moisture content. They concluded that thermal conductivity is dependent on the redistribution of moisture and thus strongly influenced by the conditions of use [32].

In the above-described research, the authors did not describe the dynamism of moisture transport in fibrous insulation materials under steady-state conditions. In this research, the author intends to compare moisture transport in loose-fill fibrous insulation materials, mineral wool, cellulose, and wood wool under steady-state conditions. In this investigation, in opposition to the above-described research, the thermal conductivity of moistened materials is not the final value but rather one of the parameters used to assess the dynamism of hygrothermal changes in materials. A novelty to the previously presented analysis of the impact of moisture on the thermal properties of insulating materials is the expression of moisture flow dynamics in materials as a reduction in the measured values of thermal conductivity in relation to the time that elapses between successive repetitions of the measurement.

## 2. Materials and Methods

### 2.1. Materials

**Figure 1:**
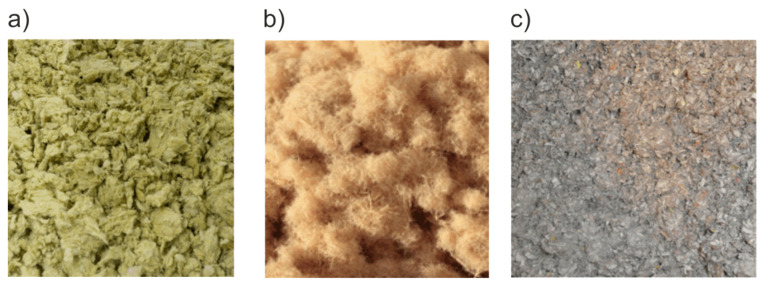
Materials in a loose state: (**a**) mineral wool, (**b**) wood wool, and (**c**) cellulose fibers.

Three loose-fill fibrous insulation materials (Figure 1) were selected for this investigation: mineral wool (MW), wood wool (WW), and cellulose fibers (CF). Their common properties are complex structures, relatively low thermal conductivity, open porosity, and susceptibility to convection. What is most important is that all of the properties change along with the changes in density. In the descriptions of the materials, pictures taken with a Vega3 Tescan (Brno, Czechia) scanning electron microscope (SEM) were used.

#### 2.1.1. Mineral Wool

**Figure 2:**
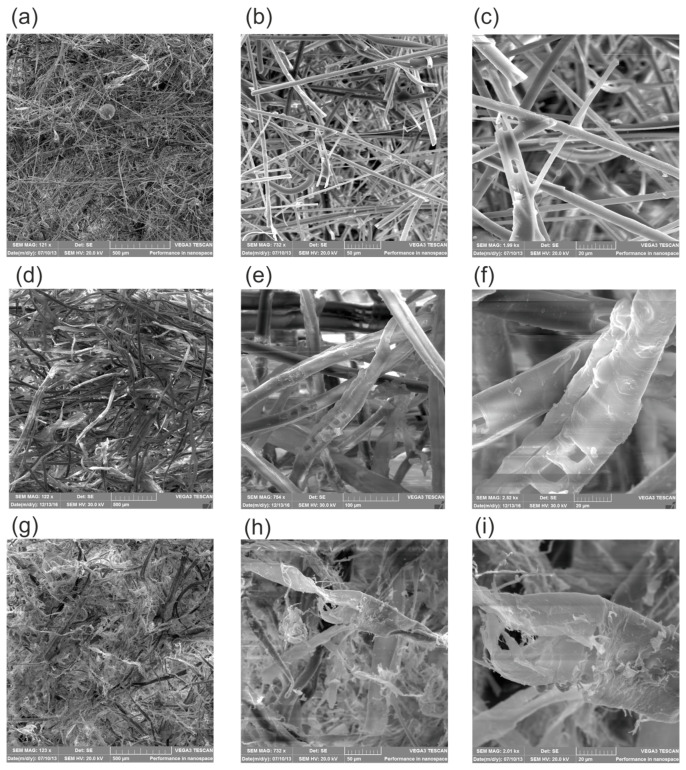
The examined materials under SEM magnification: (**a**) MW 122×, (**b**) MW 732×, (**c**) MW 1990×, (**d**) WW 122×, (**e**) WW 754×, (**f**) WW 2520×, (**g**) CF 123×, (**h**) CF 732×, and (**i**) CF 2010×.

The loose mineral wool was delivered in tightly sealed bags containing 12.0 kg ± 10%. According to the declared, intended use, this material can be used as the insulation of the above-ground parts of buildings. It is also used as the secondary filling of unheated spaces, e.g., roof slants. The producers declared a thermal conductivity coefficient of 0.039 W/(m∙K) at a bulk density of 60–70 kg/m^3^ in steady-state conditions. Although the producer did not explicitly mention it, it is possible that the mineral wool fibers were subjected to a hydrophobization process during the production stage [33]. The material is characterized by a simple construction of fibers, with a full cross-section. The observation of fibers at a magnification of 122 times (Figure 2a) exposes the multidirectional grid of thin, stiff fibers. Under a magnification of 732 times (Figure 2b), it is visible that the surfaces of the fibers are predominantly smooth and the changes in diameter over the length of the fibers are insignificant. Single fibers, not connected, are arranged in a thickened way, however multidirectional. The observation of fibers at a high magnification of 2000 times (Figure 2c) exposes the spatial grid formed by the fibers in many layers. Between the fibers with diameters of 2–10 μm, there are spaces with dimensions of 1–50 μm. The application of the conductive gold layer and its good quality during electron research indicates the stiffness of individual fibers and their low absorbability [34].

#### 2.1.2. Wood Wool

The loose wood wool was delivered in tightly sealed bags containing 15.0 kg. According to the declared, intended use, this material can be used as the insulation of the above-ground parts of buildings. Similar to loose mineral wool, it can also be used as the secondary filling of unheated spaces, e.g., roof slants. The producer declared a thermal conductivity coefficient of 0.038 W/(m∙K) at a bulk density of 35–45 kg/m^3^ in steady-state conditions. According to the manufacturer’s declaration, ammonium sulfate is added to the wood fibers at the production stage for fire protection. The material is characterized by a complicated construction of fibers. At a magnification of 122 times (Figure 2d), under the SEM, the structure of interwoven long fibers that make up wood wool is visible. Under the SEM, at a 754 magnification (Figure 2e), it is visible that the surfaces of the fibers are rough, with numerous protrusions, narrowings, and holes. Single fibers that make up the multidirectional mesh structure of the material are visible at a 2500 magnification (Figure 2f) under the SEM. This observation exposes the single fibers, which are hollow inside, thus creating a capillary system. Between the fibers with diameters of 20–35 μm, there are spaces with dimensions of 10 to 45 μm. The application of the conductive gold layer on the material surfaces reveals its medium quality during electron research which indicates the medium stiffness of individual fibers and their absorbability.

#### 2.1.3. Cellulose Fibers

The cellulose fibers were supplied in paper bags weighing 14.0 ± 0.5 kg. The cellulose fibers were recovered from newsprint and then impregnated with boron compounds against brown and light rot, to a lesser extent against blue stain and mildew, and negligible against gray rot and other passive organisms. In addition, boron, as an insecticide, is effective against typical European insect pests and also has quite good prophylactic effectiveness; to a lesser extent, it works against termites. Boron also protects the cellulose against the spread of fire. According to the intended use, the granulate can be used as fill insulation in internal and external walls, sloping, flat roofs, and ceiling structures. Cellulose fibers can also be used as a secondary filler or in unheated spaces, e.g., roof slants. This thermal insulation material is characterized by a thermal conductivity coefficient of 0.039 W/(m∙K) at a bulk density of 30–65 kg/m^3^ in steady-state conditions. Similar to the wood wool, under 123× SEM magnification (Figure 2g), the structure of a material created by the interwoven fibers is visible. What is important is that, unlike in the wood fiber structure, cellulose fibers are shorter. The SEM observation under the 722 magnification (Figure 2h) exposes the characteristic feature of cellulose, that is, the fibers create the internal capillary structure. These fibers separate in the hairline in many directions, creating larger structures which resemble the structure of plant fibers, thus confirming the natural origin of the material. Under a 2000 times microscope zoom (Figure 2i), the shape and complicated irregular fiber structure are clearly visible. The diameters of the observed fibers are in the range of 1–50 μm, while the space between the fibers is in the range of 1–150 μm. It was not possible to cover the cellulose fibers with a homogeneous layer of conductive gold. This indicates a low stiffness and rough fiber surface [34].

An observation of fibers under SEM indicates that mineral wool has the smallest fibers, with a full cross-section, and that these fibers are smooth. This distinguishes MW from cellulose and wood wool, which have jagged fibers with a capillary structure. CF have the largest distances between the fibers, which results in a greater share of convection in heat transfer and thus a higher thermal conductivity coefficient. The open capillaries of CF and WW, as well as their jagged surfaces, mean that moisture can be stored inside and on them. Thus, both natural-based fibers can achieve a higher moisture content than MW. The greatest characteristic differences between the materials are given in Table 1.

**Figure 3:**
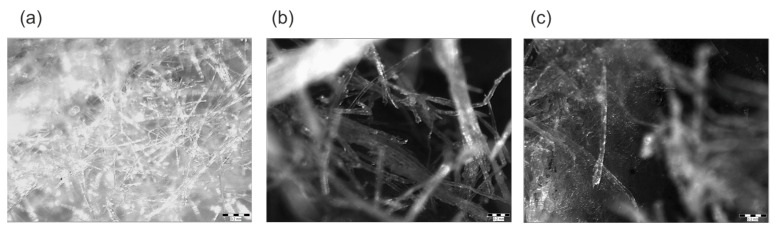
Moistened loose fibers under stereoscope magnitude: (**a**) mineral wool, (**b**) wood wool, and (**c**) cellulose fibers.

Figure 3 presents the pictures of sorption-moistened samples of selected materials. Similar to the SEM image, it can be seen that the mineral wool fibers are clearly finer. The luster of mineral wool fibers is greater than that of natural materials. This may indicate the accumulation of moisture on the mineral wool fiber surfaces. On the other hand, observation in greater magnification of cellulose and wood wool, unlike mineral wool, indicates the presence of small water particles inside the fibers. Unfortunately, it was not possible to record these images with a camera.

### 2.2. Sample Preparation

**Figure 4:**
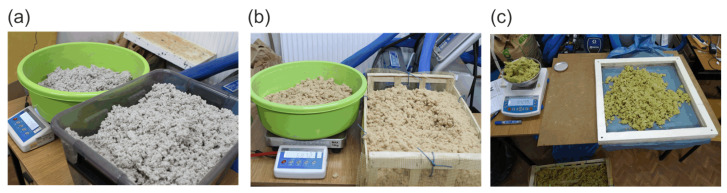
Test sample preparation, random selection of fibers, and weighing: (**a**) cellulose fibers, (**b**) wood wool, and (**c**) placing the fibers of mineral wool in frame.

Loose, fragmented materials were stored in isothermal conditions at approximately 20 °C over distilled water in airtight boxes. Conditioning lasted around one month, and the materials were often moved and mixed to achieve an even moisture distribution. Sample preparation consisted of a random sampling of fibers from the conditioned material, grinding it, and placing it evenly in a frame made of extruded polystyrene (Figure 4). The 45 mm thick frame, with internal dimensions of 510 × 525 mm, was covered on both sides with a thin foil and sealed by plastic tape to immobilize the tested bulk materials.

**Figure 5:**
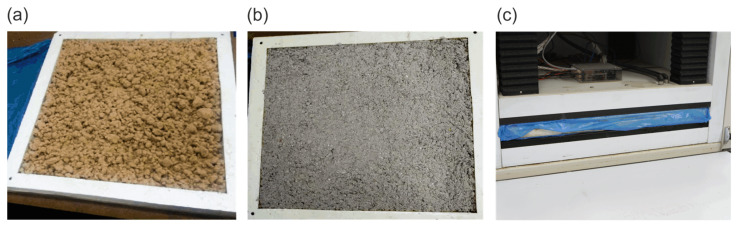
Frames filled with loose materials before sealing with foil: (**a**) wood wool and (**b**) cellulose fibers. (**c**) Frame with mineral wool in the HFM Fox 602 apparatus.

For each sample preparation, the prepared frame with the material inside was immediately placed in the HFM Fox 602 apparatus manufactured by Laser Comp (Saugus, MA, USA). Attempts were made to prepare a sample with a constant weight of 1100 g for mineral wool and 1000 g for cellulose, with a tolerance of up to 2 g. For wood wool, samples of various weights were prepared in the range of 525–694 g. The difference in the weight of the moistened material affected the difference in density concerning the weight of the dried material. Finally, four samples of wood wool (density range 36.2–47.0 kg/m^3^), four samples of mineral wool (density range 90.6–91.4 kg/m^3^), and five samples of cellulose (density range 69.6–76.1 kg/m^3^) were prepared. Figure 5 presents formed samples before or during the measurements. The densities refer to the weight of the dried materials.

### 2.3. Method

Before the measurements in the HFM apparatus, the moisture content of the tested materials was gravimetrically measured by taking samples from their representative fibers just before the test. In addition, for 3 out of 5 tested samples, two samples of cellulose fiber materials were taken into consideration after the test from the upper and lower parts adjacent to the warm and cold plates, respectively. In the case of wood wool, the weight of the sample was tested before and after the test. The moisture absorption was calculated from the weight gain which was determined constantly using a laboratory drier and precise Radwag AS310/X balances with 0.1 mg accuracy manufactured by Radwag (Radom, Poland).

**Figure 6:**
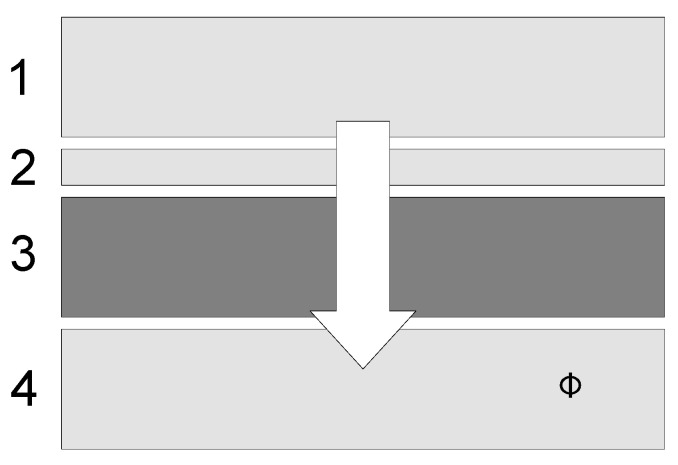
A sketch of HFM Fox 602 adopted for conductivity test: 1–heating plate, 2–heat flux density sensor, 3–sample, and 4–cooling plate, Φ—heat transfer [W].

The HFM FOX 602 was utilized to determine the thermal conductivity of flat insulation in a steady state based on the international standard EN 12667 [35]. The measuring range of thermal conductivity was from 0.002 to 2 W/mK. The measurement error resulting from the method was specified in the standard as 6%, and the maximum probable error was 2.4%. The test consisted of creating a constant temperature field on the upper and lower plates by the plate apparatus (in this case, the temperature of the upper plate was set at 20 °C and the lower plate at 0 °C) and the registration of the heat flux flowing through the tested sample placed in the measuring chamber. The Fox 602 instrument operates on the basis of a one-dimensional Fourier law. A sketch of the apparatus is presented in Figure 6.

The tests consisted of carrying out measurements of the thermal conductivity of the selected materials. During the tests, moisture contained in the materials naturally moved one-directionally toward the cold plate, where condensation could occur. This is consistent with the conclusions of Peuhkuri’s thesis [36] and Simonson et al.’s work [37]. The time between measurement repetitions for each sample was also recorded for analysis. Thirteen repetitions for each tested sample were conducted. Assuming a sample thickness of 45 mm and protective foils, the distance between the plates was set at 47 mm with a tolerance of 0.2 mm. Differences in the position of the boards within the indicated range meant that the tested samples within one measurement cycle slightly differed in density (for mineral wool, the differences were 0.18–0.39 kg/m^3^; for cellulose, the differences were 0.27–0.39 kg/m^3^’ for wood wool, the differences were up to 0.09–0.14 kg/m^3^).

The measurement procedure was as follows. During each measurement, 512 readings were collected in one block. Within the block, data on the temperature of the upper and lower plates and signals from transducers mounted in the upper and lower plates were collected. The collected data were compared with the corresponding averaged values from the previous measurement block. When the comparison met the steady state conditions, the system considered the measurement complete.

Analysis of moisture content changes, the distribution of measured thermal conductivity values, and the speed of processes resulted in a comprehensive analysis of moisture flow in the selected fibrous materials.

## 3. Results

### 3.1. Moisture Content of Tested Samples

Table 2 presents the moisture content of the tested MW in the range from 0.09 to 0.39%. On the one hand, these are very low results, below the measured sorption moisture content (0.83%) [34]. On the other hand, the sorption measurements were conducted in desiccators, while in the presented work, several kilos of material had to be conditioned over the demineralized water. This may produce a significant difference.

Table 3 presents the moisture content of the tested CF before the HFM measurements, in addition to, for samples 1–3, the moisture content after the test was measured separately for the upper and lower fibers. The moisture content of samples before the tests was in the range from 8.20 to 15.73%, which is below the measured sorption values in the desiccators (41.76%) [34]. Similarly, as in the case of mineral wool, the large volume of the material prevented achieving a higher (or equal) moisture content.

In this case, however, it is visible that moisture tests after the HFM tests clearly indicate the transport of moisture in the material toward the cold bottom plate. After the tests, the moisture content of the lower fibers were higher than the upper ones by 3.90× for sample 1, 5.28× for sample 2, and 4.09× for sample 3. In each case, the moisture content of the upper fibers after the tests were lower than the initial values, while that of the lower fibers was higher by 0.47 to 2.70×.

Table 4 presents the moisture content of loose WW before and after the HFM tests. Almost uniform moisture content in the material (19.91–19.92%) was achieved. It was lower than in the sorption tests (65.28%) [34]. It is also significant that after the tests, the moisture content for all samples decreased to the level of 15.83 to 18.01%. The lowest moisture content was for sample 3, and the highest was for sample 1. Importantly, there was no direct relationship between the tested sample mass and the moisture content measured after the test.

The author notes that uniform moisture content of the tested samples was not achieved, even though the materials were systematically crushed and mixed to ensure the most even conditions. These were large portions of materials, several kilograms each. What is more, loose fibrous materials may exhibit different properties after the preparation of the samples. One sample may contain more shorter fibers, while another may have longer fibers or include more dust, which may affect the moisture content achieved in a prepared sample. In the case of mineral wool, the variation in moisture content is large, but these are all values below 0.4% moisture content. In the case of wood wool, a constant moisture level was achieved. In the case of cellulose fibers, the diversity of moisture was the greatest, which may result from the clumping of this material in humid conditions.

### 3.2. Thermal Conductivity Stabilization Time

**Figure 7:**
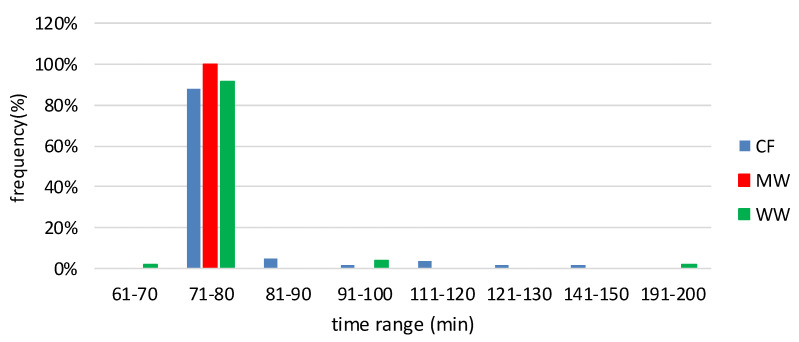
Thermal conductivity stabilization time of MW, WW, and CF samples.

Thermal conductivity stabilization time was adopted as a criterion for assessing the repeatability of the characteristics of moistened materials placed in steady-state conditions. As described in Section 2.3, every measurement consisted of a big number of signal readings. When the comparison of signals met the steady state conditions, the system considered the measurement complete. The repeatability of the duration of subsequent measurements means that the moisture state of the material between the readings does not change significantly, so the moisture transport is not intensive. The results of stabilization time are presented in Figure 7.

The longer it takes to reach steady-state conditions during measurement means that moisture transport is more intense. Thermal conductivity stabilization time in the case of MW lasted 72–73 min. In the case of WW and CF, more than 85% of the registered times were also 72–73 min, but there were also other results, except in one case, of longer times. Assuming that the repeatability of the time needed to perform the thermal conductivity measurement is an indicator of material testing stability, the highest stability was for mineral wool, while the lowest was for cellulose fibers.

### 3.3. Thermal Conductivity

#### 3.3.1. Mineral Wool

**Figure 8:**
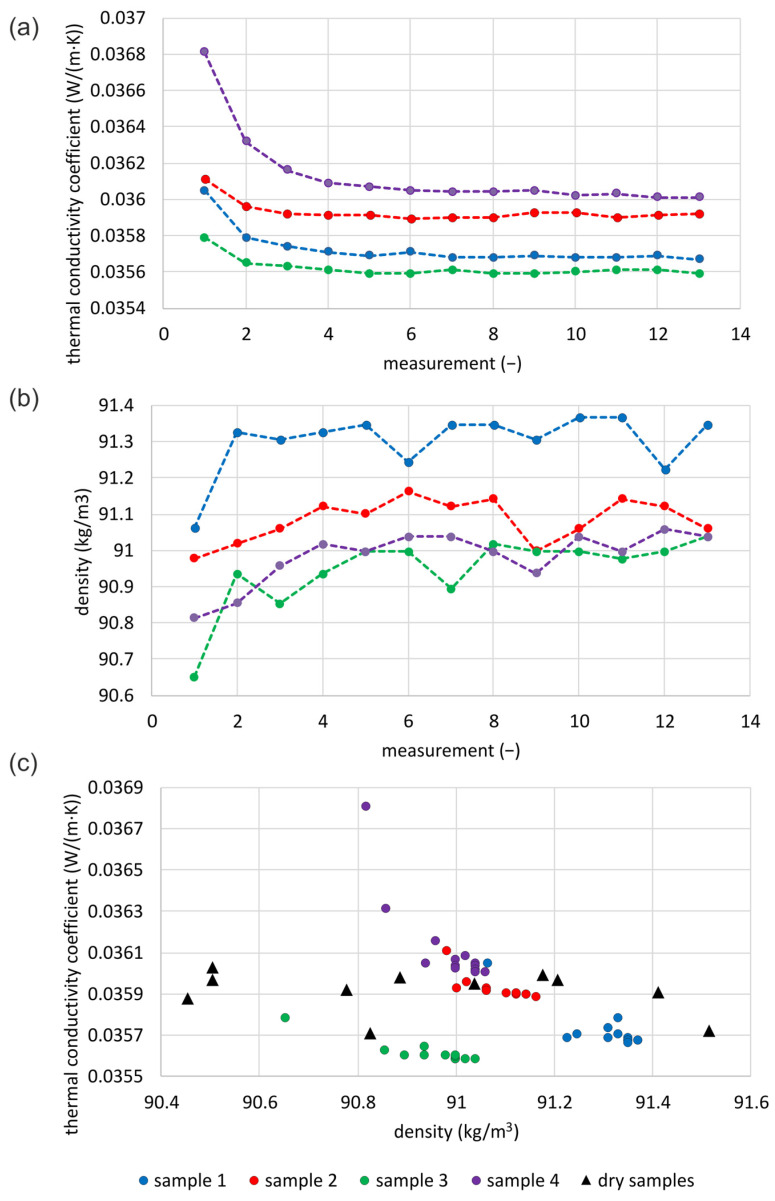
Examination of moistened loose MW: (**a**) thermal conductivity of samples, (**b**) densities of tested samples, (**c**) comparison of thermal conductivity with dry samples of similar densities.

Figure 8a presents the results of the thermal conductivity of loose MW. The graph shows 13 measurement results for each of the four samples. The first measurement for each of the samples resulted in the highest thermal conductivity, and then it decreased to stabilize from the fifth measurement. The differences in measured thermal conductivity between the first and second measurements of each sample are small, with a maximum of 1.4%. The first readings correspond to the lowest recorded density values (Figure 8b). In the second measurement, the densities of the samples are higher by 0.04–0.28 kg/m^3^, which is up to 0.3% of the sample base densities. From the second measurement, the differences in density are smaller. These differences in densities are due to a set tolerance of 0.2 mm for the position of the boards. There are probably several reasons as to why the plates were not evenly positioned: shaking the sample while lifting the plate before the next measurement, the presence of foil, and the possible formation of an air cushion above the sample under the foil.

Figure 8c presents a comparison between the results achieved for the tested moistened samples and the tested dry samples for a similar range of densities [38]. Observation of the results may lead to the conclusion that the values do not differ from each other by more than 1% (except for the extreme sample for the fourth series), which is below the measurement error of the device. Different results could have arisen due to differences in the thickness of the tested samples, the degree of mineral wool fragmentation, or the influence of external conditions. The results of series two (0.29% moisture) are the closest to the results obtained for dry samples.

For each tested sample, linear correlations (Table 5) of the measured results of thermal conductivity with the number of repetitions of the measurement and with the density of the tested material were examined. The correlation with the number of measurement repetitions is in the range of 0.261–0.478. The highest correlation corresponds to sample no. 4, for which the highest values of thermal conductivity were recorded. The lowest correlation corresponds to sample no. 2. The correlation of the results with the density of the tested material in subsequent repetitions is within the range of 0.556–0.871. The highest correlation with density occurs for sample no. 3 with the measured lowest density and, at the same time, the lowest value of the thermal conductivity coefficient. The smallest correlation with density again corresponds to sample no. 2.

In the next step of the correlation analysis (Table 5), the first measurement readings are deleted. The reason is that they differed greatly from the rest. The range of both determined correlations has expanded. In the correlation with the number of repetitions of the measurement, the lowest value of 0.065 (4 times lower) is calculated again for sample no. 2. The highest value of 0.609 (about 1.5 times higher) again for sample 4. The smallest linear correlation with a density of 0.046 is for sample no. 1, which in the previous analysis reached a value of 0.716. The highest correlation of 0.782 is achieved by sample no. 2. After omitting the first measurement, the correlation with the number of repetitions increases significantly for two measurements, slightly decreases for one, and significantly decreases for one. The correlation with the sample density decreases in three cases.

#### 3.3.2. Cellulose

**Figure 9:**
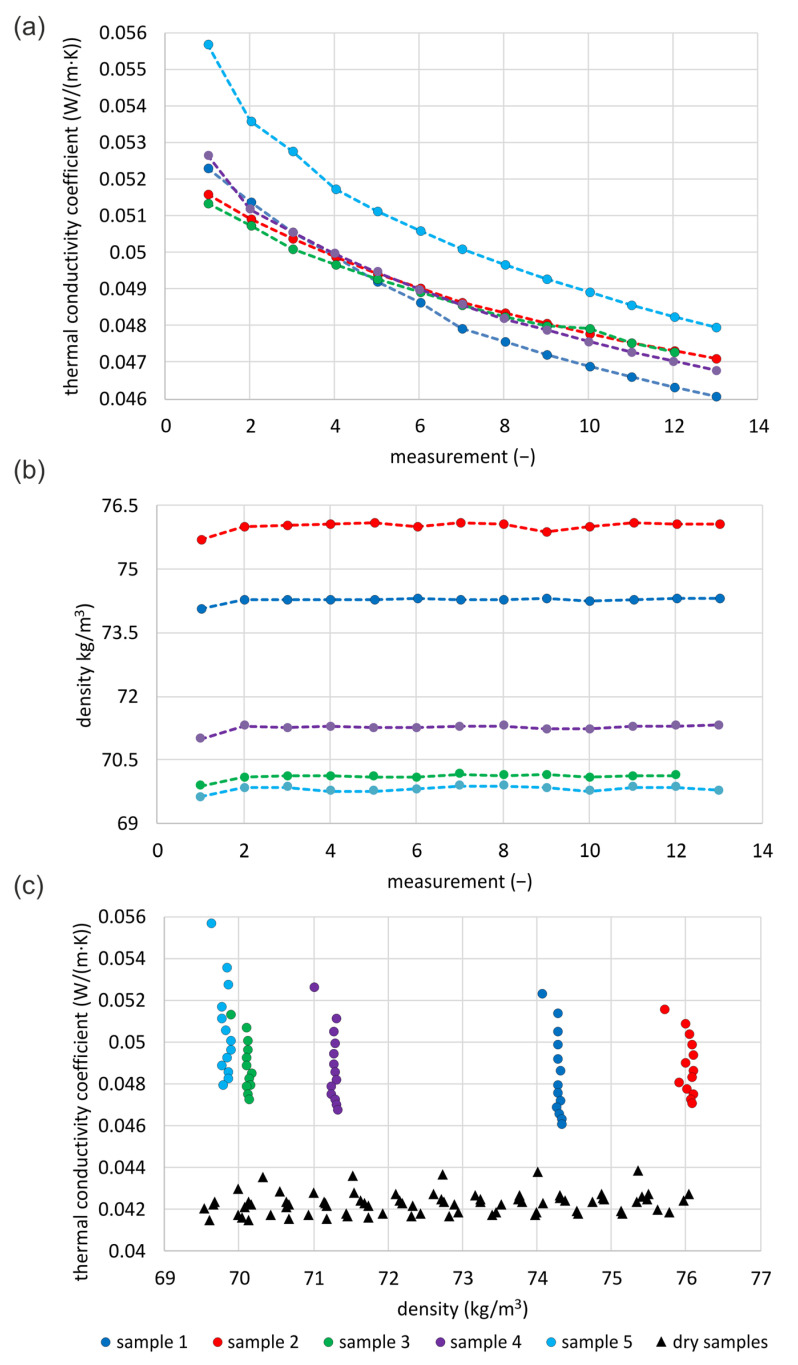
Examination of moistened CF: (**a**) thermal conductivity of samples, (**b**) densities of tested samples, (**c**) comparison of thermal conductivity with dry samples of similar densities.

Figure 9a presents the results of the thermal conductivity of CF. The graph shows 13 measurement results for four samples and 12 for the third sample. Since the samples differed in moisture content, every time a constant mass of them was taken for the measurement, they finally differed in density related to the dry material. Hence, Figure 9b shows the densities of the tested samples in two ranges. Samples 1 and 2 are characterized by lower moisture content, so their densities related to the dry material are higher. As with loose mineral wool samples, the first thermal conductivity readings correspond to the lowest density of the individual samples. What is different from mineral wool is that the densities are almost stabilized from the second measurement. A steady trend of decreasing thermal conductivity measurements can be seen for all tested samples while stabilizing the density of the samples.

The difference between the first and second measurements of the thermal conductivity is the highest in the case of sample no. 5 (4.0%). This sample has the highest moisture content and lowest density. The second highest (2.9%) is in the case of sample no. 4. In the case of sample no. 3 (second highest moisture content), it is 1.2%, while for the lowest moistened samples, no. 1 and 2, it is 1.8% and 1.3%. The density differences between the first and second measurements are 0.20–0.30 kg/m3, which is up to 0.4% of the difference in density. From the second measurement, these differences are smaller. The reasons as to why different material densities occur within one prepared sample may be similar to the mineral wool case. Finally, the difference in measured thermal conductivity between the first and twelfth measurements (unified due to sample no. 3) for the first sample is 11.5%, for the second sample—8.3%, for the third sample—7.9%, for the fourth—10.7%, and for the fifth sample—13.4%. These values are higher than the measurement error of the method. The highest difference is obtained for the sample with the highest moisture content and the lowest density, but the smallest difference is obtained for the second highest moisture content, sample no. 3.

Figure 9c presents a comparison between the measured values of thermal conductivity of the tested samples and the dry samples of similar density range [39]. The differences in the results reach up to 13%. In the case of moistened materials, results below 0.046 W/mK are not achieved (sample 1 was the closest), while for dry samples, the highest values are up to approximately 0.044 W/mK.

For each tested sample, linear correlations (Table 6) of the measured results of thermal conductivity with the number of repetitions of the measurement and with the density of the tested material are examined. The correlation with the number of measurement repetitions is in the range of 0.918–0.971. The lowest correlation is for the fifth sample, with the highest moisture content (and lowest density), and the highest is for the second sample, with the lowest moisture content. The correlation between thermal conductivity and density is linear and falls within the range of 0.267–0.452. The sample with the highest humidity (fifth sample) has the lowest correlation, while the first sample has the highest correlation. The second most dense sample also shows a relatively high correlation.

In the next step of the correlation analysis (Table 6), the first measurement readings are deleted. The reason is that they differed greatly from the rest. The range of both determined correlations changed. The linear correlation of thermal conductivity with the number of repetitions is in the range of 0.961–0.978 and increases for all cases. The lowest is for the lowest density sample no. 5, and the highest is for the highest density sample no. 2. The linear correlation of thermal conductivity with the density of the tested samples decreases significantly. For sample 1, the second densest, there is a reduction of 60%, but for the others, it is at least 280%, and for sample 5, it is more than 300 times.

#### 3.3.3. Wood Wool

**Figure 10:**
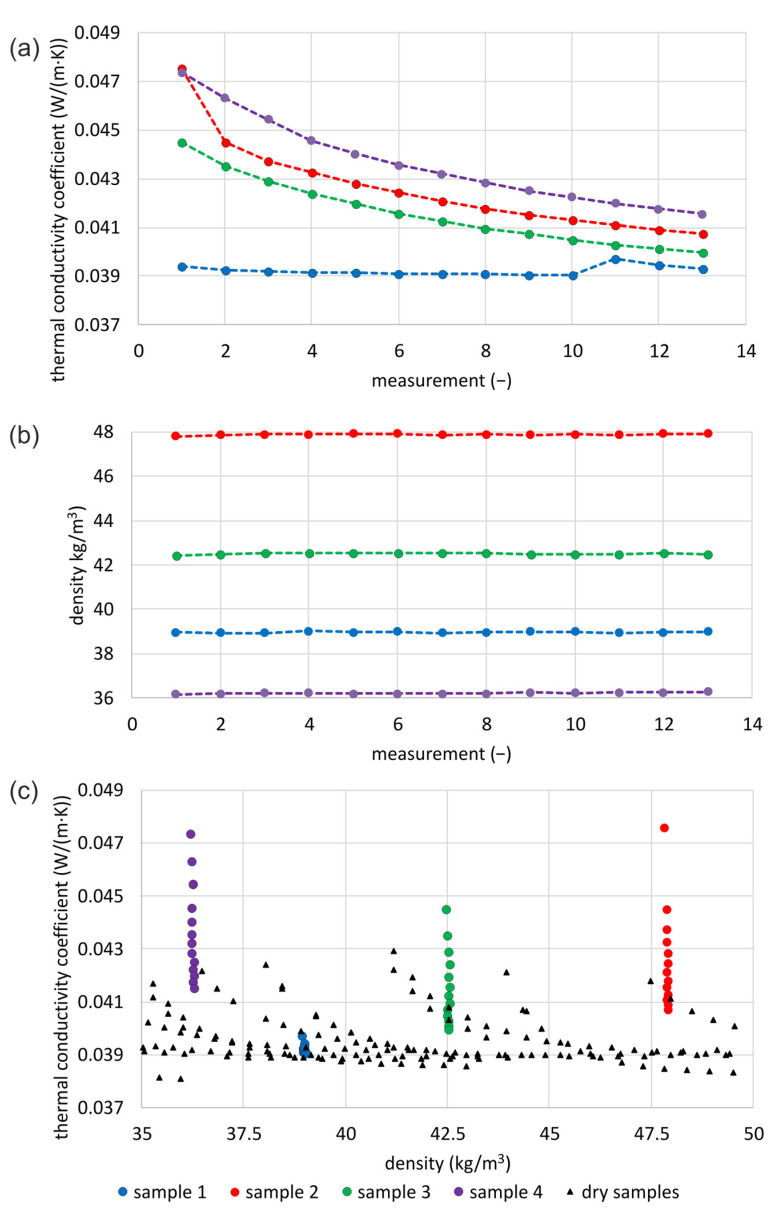
Examination of moistened loose WW: (**a**) thermal conductivity of samples, (**b**) densities of tested samples, (**c**) comparison of thermal conductivity with dry samples of similar densities.

Figure 10a presents the results of the thermal conductivity of loose WW. The graph shows 13 measurement results for each of the four samples. Figure 10b presents the densities of the tested samples. The lowest density is for sample no. 4, and the highest is for sample no. 2. In the case of sample no. 1, the first density tested is neither the highest nor the lowest, but is higher than the next two. For other samples, the density of the first reading is lower than the following ones. As in the case of cellulose fibers, a constant trend of a decreasing value of the measured thermal conductivity with successive measurements can be observed. The exception is sample no 1, in which such a trend includes up to ten repetitions, although the last three results deviate from this tendency. However, even for sample no. 1, the first conductivity reading is larger than the following (except replicates 11 and 12). For the tested samples, the first value is higher than the second by 0.4% for the first sample, 6.5% for the second sample, and 2.2% for the third and fourth samples. At the same time, the density of the samples tested in the second reading is lower in the case of the first sample by 0.17 kg/m^3^, for the second sample higher by 0.06 kg/m^3^, for the third sample higher by 0.04 kg/m^3^, and for the fourth sample higher by 0.03 kg/m^3^. These differences range from 0.04 to 0.13%. The difference between the first and last thermal conductivity measurements is 0.2% for the first sample, 14.4% for the second sample, 10.2% for the third sample, and 12.3% for the fourth sample. This means that the greatest difference is for the sample with the highest density, the second for the lowest density sample, the third for the second most dense sample, and finally, the smallest for the third most dense, sample 1.

Figure 10c presents a comparison between the measured results of thermal conductivity for the tested samples and the dry samples tested for a similar density range [40]. The large visible range of measured values for samples 2–4 and the concentration of the values for sample 1 are characteristic. The results of sample 1 are within the presented range of the results of the dry samples. For samples 2–4, the last measured values overlap the range of dry sample results, but at least half of them deviate significantly. However, this is a smaller difference than in the case of cellulose fibers.

For each tested sample, linear correlations (Table 7) of the measured results of thermal conductivity with the number of repetitions of the measurements and with the density of the tested material are examined. The correlation with the number of measurement repetitions is in the range of 0.059–0.929, but for three samples, it is over 0.802. The lowest correlation is for the first sample, in which the course of the measured values is significantly different from all others in the entire study. The highest correlation is for the third sample, the second most dense. The correlation with density is in the range of 0.190–0.564. The smallest correlation is for the third sample, and the highest correlation is for the second sample (with the highest density).

In the next step of the correlation analysis (Table 7), the first measurement readings are deleted. The reason is that they differed greatly from the rest. Changes then occur in both examined linear correlations. The linear correlation of thermal conductivity with the number of measurement repetitions ranges from 0.177–0.959 and increases for all cases. The lowest remains for the first sample, and the highest is for the second and third samples. The linear correlation of thermal conductivity with the density of the tested samples also decreases significantly. It increases by 3% only for the first sample, while it decreases for the remaining cases, in the case of the third sample, by as much as 37,980 times.

## 4. Discussion

In the described study, MW achieves lower values of sorption moisture over demineralized water than WW and CF. Moreover, the tests of the thermal conductivity of loose mineral wool showed that the results stabilized quite quickly. Of course, as described in the introduction, such stabilization does not mean that a full steady-state equilibrium has been achieved. Nevertheless, this material presented different abilities than CF and WW.

Based on the results obtained from this study, it was observed that the thermal conductivity measurement of mineral wool exhibited a consistent trend, with the first measurement always being higher than the second by a margin of 0.4% to 1.4%. However, for two samples, the results only achieved stability after the fifth reading, while for the other two samples, there were still small differences in percentage between successive readings.

In the case of CF, the first reading showed a higher thermal conductivity measurement by 1.2% to 4.0% when compared to the second reading. However, there was no stabilization of the results throughout the research cycle, with each subsequent reading showing a lower thermal conductivity measurement than the previous one.

It can be concluded that this is a constant decreasing trend with the repetitions of the measured thermal conductivity. In the case of wood wool, the first reading was also higher for each sample compared to the second reading by 0.4–6.5%. For one of the samples, the following measurements did not follow a clear downward trend, while for the others, this trend is visible.

As shown in Figure 3, the MW becomes wet on the surface of its fibers. The fibers have a full cross-section, so moisture absorption cannot take place inside them. It is different in natural materials—cellulose fibers and wood wool. Their fibers are larger, hollow inside, and allow moisture to be stored inside and on the surface of the fibers. The differences in the structure of the materials, and thus the difference in the method of moisture accumulation, affect their predisposition to moisture as a result of sorption and then also the transport of moisture through their structure. The measured initial moisture content of the mineral wool was very low, about half of that in the standard sorption test. For this reason, no moisture content tests were performed after the measurement. It was assumed that the stabilization of the results in HFM means that a quasi-steady state has been reached. The measured initial moisture content of the cellulose fibers and wood wool was also lower than for the standard tests but much higher than for mineral wool.

The moisture content of the CF located close to the cold and warm plates, measured after the HFM tests, clearly indicated the movement of moisture toward the cooler plate. However, even the ones adjacent to the warm plate, after about 15 h of HFM testing, were still damp. The moisture content of WW before and after the HFM measurement showed that the fibers lost only a few percent of their moisture content. This means that the transport of moisture in natural materials is a much longer process than in mineral wool. The HFM apparatus is not a hermetically sealed device, and moisture loss through the samples is possible during testing.

**Figure 11:**
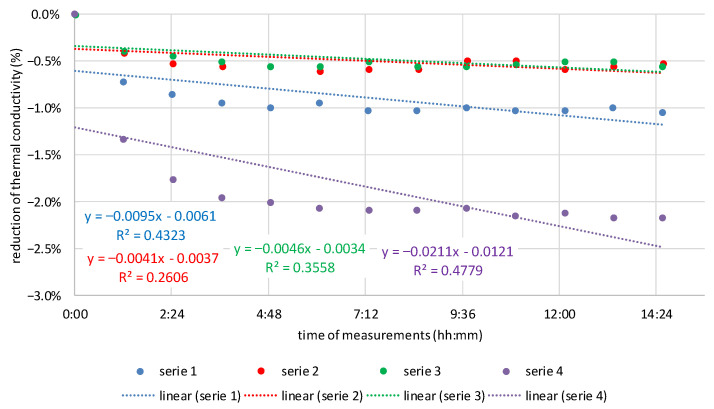
Moisture flow dynamics in loose MW expressed as a reduction in the measured values of thermal conductivity in relation to the time that elapsed between successive repetitions of the measurement.

**Figure 12:**
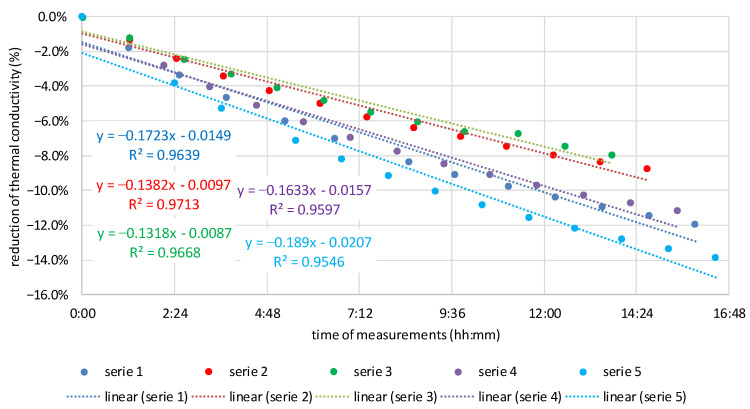
Moisture flow dynamics in CF expressed as a reduction in the measured values of thermal conductivity in relation to the time that elapsed between successive repetitions of the measurement.

**Figure 13:**
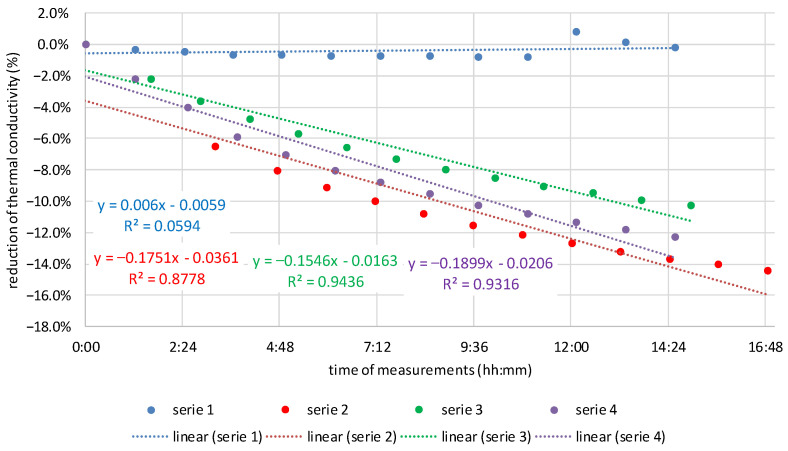
Moisture flow dynamics in loose WW expressed as a reduction in the measured values of thermal conductivity in relation to the time that elapsed between successive repetitions of the measurement.

Assuming that the transport of moisture in the insulation material changes its thermal conductivity, it was decided to investigate the dynamics of these changes. Along with the increase in moisture flows, the dynamics of changes in the thermal conductivity of the tested materials also increased. The angle of inclination of the trend line to the *X*-axis was adopted as the criterion for assessing the dynamics of changes. The greater the slope angle, the greater the dynamics of change, and this means greater moisture flow. Figure 11, Figure 12 and Figure 13 show the dynamics of thermal conductivity reduction for MW, CF, and WW. It is expressed as a percentage reduction in the measured values of thermal conductivity in relation to the time that elapsed between successive repetitions of the measurement for a given sample.

Based on the results presented in Figure 11, Figure 12 and Figure 13, it can be seen that the smallest dynamics of changes are for mineral wool, for which the slope of the linear regression line is between −0.008 and −0.033. For cellulose fibers, the range of slope is from −0.141 to −0.210. For wood wool, for the first measurement, the slope is 0.001, but for the others, it is from −0.162 to −0.211. Apart from the first sample of wood wool, the slopes of the linear regression line for both materials of natural origin are similar and definitely higher than for mineral wool.

One sample in the case of MW and WW does not show linearity. These results were excluded from the conclusions. However, they were left in the presentation to demonstrate the complexity of laboratory tests. From the analysis of slopes of the linear regression line, it can be concluded that in natural materials there is a greater movement of moisture than in MW. The dynamics of thermal conductivity reduction in CF and WW samples placed in steady-state conditions is several times higher than in MW. This means that more moisture flows in these materials within a similar time as in MW, and therefore, this transport is more intense. This may be a good indication for further analysis of moisture buffering in natural materials.

The average deviation values were determined for the recorded measurement time of individual results (Table 8). The lowest standard deviation values were determined for mineral wool measurements and the highest for cellulose measurements. For wood wool measurements, the exception is sample 1, and the other samples also have high deviations. This shows that the measurements for mineral wool are more stable, which means that moisture is quickly removed from the material. In contrast to this are the results obtained for cellulose fibers and wood wool. In these materials, moisture transport, although it is more intense, is long lasting. It is associated with the removal of moisture from inside the fibers. This process is hindered by the air present in the pores.

**Figure 14:**
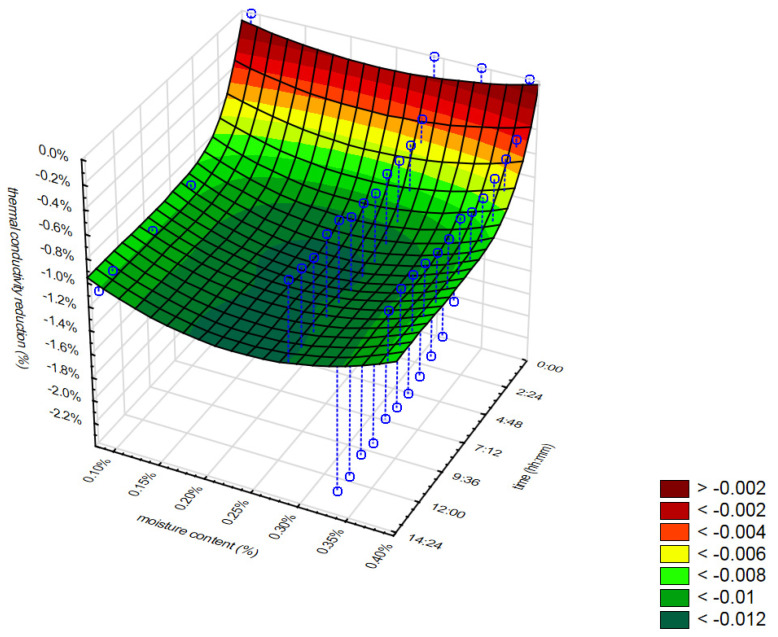
Relation between thermal conductivity reduction, moisture content, and time of measurements for MW.

**Figure 15:**
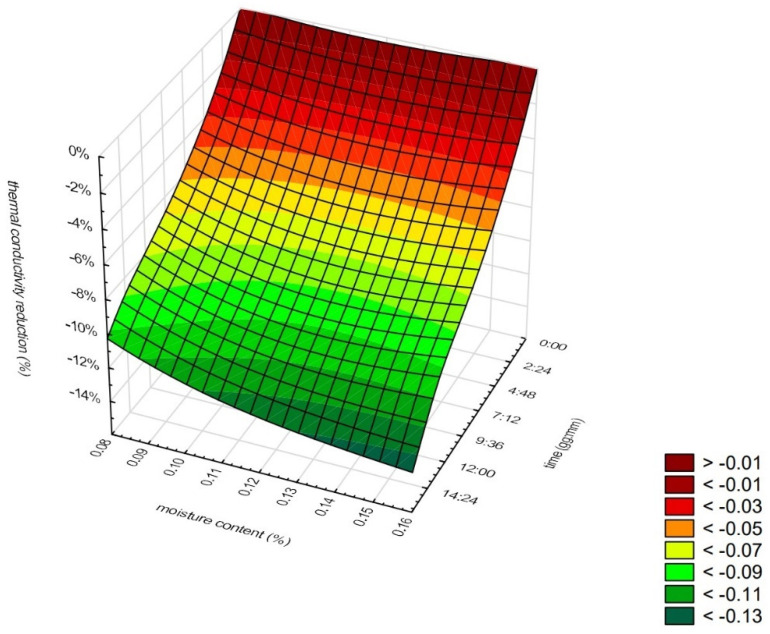
Relation between thermal conductivity reduction, moisture content, and time of measurements for CF.

**Figure 16:**
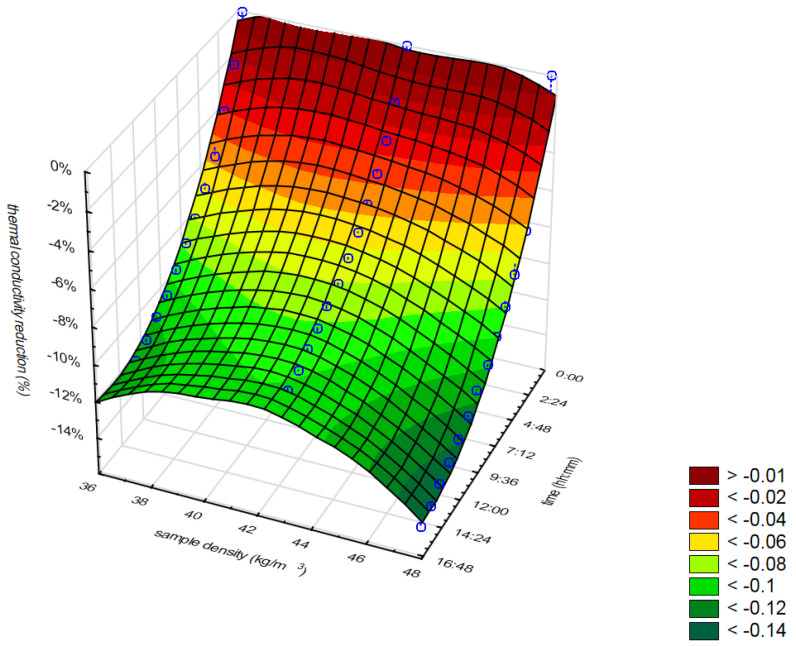
Relation between thermal conductivity reduction, moisture content, and time of measurements for WW.

Figure 14 and Figure 15 present the relation between the thermal conductivity reduction, moisture content, and time of measurements for mineral wool (Figure 14) and cellulose fibers (Figure 15). Figure 16 presents the relation between the thermal conductivity reduction, sample density, and time of measurement for WW. Additionally, it is important to note that the results presented in Figure 16 do not include the data for the first sample, which was an exceptional case. In this case, the moisture content of the samples was almost equal.

As can be seen in Figure 14, the reduction in the thermal conductivity is low within the first 4 h of measurement, and after that, the area is almost flat. It is visible that moisture content does not affect thermal conductivity in the tested range. The reduction in moisture content for the samples of the lowest and highest moisture content is almost the same. The minor discrepancies observed in the thermal conductivity measurements of loose mineral wool suggest that the material may have experienced moisture loss over a brief period. In the case of cellulose fibers (Figure 15), the reduction in the thermal conductivity is rapid during the whole tested time. It can be seen that the more moistened the samples were, the faster and the higher the thermal conductivity reduction was.

In the case of WW (Figure 16), the initial moisture content of the samples was almost equal. This enables an analysis of the density impact on moisture transport within the samples. The most rapid is the thermal conductivity reduction in the densest sample. This sample contains the most moisture.

The 3D analysis shows that in the more moistened samples, the moisture transport is greater and faster.

## 5. Conclusions

Moisture flow in fibrous materials is a complex phenomenon. First of all, it depends on the structure of the fibers of the material. The presence of large fibers, which are hollow inside (natural materials), favors the accumulation of moisture in them. Moisture flow in such materials takes place both in the pores between the fibers and inside the fibers. The surface of wood wool and cellulose fibers is not smooth or straight. As a result, it has a greater ability to accumulate moisture than materials with slippery fibers, such as mineral wool. As a result, natural materials achieve higher sorption moisture than synthetics.

In materials with smooth and filled fibers, such as mineral wool, the moisture flow takes place only in the pores between the fibers.

This study on the dynamics of changes in the thermal conductivity of sorption wet materials was intended to indicate which factors mainly affect the transport of moisture in fibrous insulation. The method of wetting the materials by sorption was chosen as purposeful. Treating materials with water droplets could make the moisture distribution very random. In the case of mineral wool, which reached a low content of sorption moisture, stabilization of the measurement was fast. The measured thermal conductivity after a few hours of the test was comparable to that of the dry material. However, for cellulose fibers and wood wool, the conductivity of the wet materials was higher than that of the dry materials. These materials reached higher sorption moisture. A dynamic analysis of changes in thermal conductivity shows the following:

1. Moisture transport in materials of natural origin is not uniform.
2. Moisture transport in fibrous materials depends on the moisture content of the material. The wetter the material, the faster the transport.
3. The presence of air in the pores affects the velocity and mass of moisture transferred.
4. A sorption moisture content not exceeding 0.4% in the case of WM leads to an increase in thermal conductivity of 1%. After about 6 h of constant temperature difference conditions, stabilization took place and the thermal conductivity of the tested samples was comparable to that of the dry material. In the case of CF, a sorption moisture content of 8.2 to 15.7% leads to an increase in thermal conductivity of 29%. After about 14–16 h of constant conditions, the temperature difference does not stabilize, but the thermal conductivity is already 14% higher than that of the dry samples. In the case of WW, a sorption moisture content of 19.9% leads to an increase in thermal conductivity of 16%. Similar to cellulose, stabilization does not take place after about 14–17 h, but the thermal conductivity is only 1% higher compared to the dry samples.

Among the applications where the influence of moisture on thermal conductivity should be taken into account is the initial period of use of buildings, especially frame structures. In many countries, insulation works are also carried out during periods of increased air humidity, which contributes to the sorption of fibrous insulation. Operational problems of moisturizing the insulation built into the building elements related to the lack of tightness of the vapor retarder are also significant. Then, however, the greater effect on the moisture content of fibrous insulations is moisture diffusion rather than sorption.

## Figures and Tables

**Table 1:** Comparison between MW, WW, and CF based on the SEM observation.

	Mineral Wool	Wood Wool	Cellulose Fibers
Fiber diameter	2–10 μm	20–35 μm	1–50 μm
Space between the fibers	1–50 μm	10 to 45 μm	1–150 μm
Fibers’ capillarity	no	yes	yes
Fibers’ surface	smooth	jagged	jagged

**Table 2:** Moisture content of tested MW.

Number of Sample (−)	Sample Mass (g)	Moisture Content of the Sample (%)
1	1102.03	0.09
2	1102.48	0.29
3	1102.14	0.39
4	1102.30	0.34

**Table 3:** Moisture content of tested CF.

Number of Sample (−)	Sample Mass (g)	Moisture Content of the Sample (%)		
		Before Tests	After Tests, Upper Fibers	After Tests, Lower Fibers
1	1000.27	10.36	3.91	15.25
2	1000.00	8.20	4.20	22.18
3	1001.77	15.45	6.93	28.34
4	1000.00	13.98	-	-
5	1000.00	15.73	-	-

**Table 4:** Moisture content of tested WW.

Number of Sample (−)	Sample Mass (g)	Moisture Content of the Sample (%)	
		Before HFM Tests	After HFM Tests
1	565.0	19.92	18.01
2	694.0	19.91	17.85
3	616.0	19.92	15.83
4	525.0	19.92	17.86

**Table 5:** Linear correlation of thermal conductivity of loose MW with the number of repetitions and density of samples.

Linear Correlation of Thermal Conductivity, Sample Number	Number of Repetitions		Density of Tested Samples	
	All Readings	Without the First Reading	All Readings	Without the First Reading
1	0.433	0.601	0.716	0.046
2	0.261	0.065	0.556	0.782
3	0.356	0.295	0.871	0.501
4	0.478	0.609	0.752	0.741

**Table 6:** Linear correlation of thermal conductivity of CF with the number of repetitions and density of samples.

Linear Correlation of Thermal Conductivity, Sample Number	Number of Repetitions		Density of Tested Samples	
	All Readings	Without the First Reading	All Readings	Without the First Reading
1	0.958	0.962	0.452	0.268
2	0.971	0.978	0.268	0.009
3	0.967	0.974	0.434	0.155
4	0.943	0.977	0.369	0.005
5	0.918	0.961	0.267	7.00 × 10^−5^

**Table 7:** Linear correlation of thermal conductivity of loose WW with the number of repetitions and density of samples.

Linear Correlation of Thermal Conductivity, Sample Number	Number of Repetitions		Density of Tested Samples	
	All Readings	Without the First Reading	All Readings	Without the First Reading
1	0.059	0.177	0.315	0.325
2	0.802	0.959	0.508	0.054
3	0.936	0.959	0.190	5.00 × 10^−6^
4	0.929	0.942	0.564	0.406

**Table 8:** The average deviation values for the recorded measurement time of individual results.

Number of Sample (−)	Number of Repetitions		
	MW	CF	WW
1	3.14 × 10^−4^	8.74 × 10^−3^	2.70 × 10^−4^
2	2.70 × 10^−4^	1.27 × 10^−3^	2.38 × 10^−2^
3	3.14 × 10^−4^	3.27 × 10^−3^	4.65 × 10^−3^
4	3.14 × 10^−4^	1.09 × 10^−2^	1.80 × 10^−3^
5		1.59 × 10^−2^	

## Data Availability

The data presented in this study are available on request from the author.
